# Parasite Fauna of the Dusky Grouper (*Epinephelus marginatus,* Lowe 1834) from the Central Mediterranean Sea

**DOI:** 10.3390/ani11092523

**Published:** 2021-08-28

**Authors:** Giovanni De Benedetto, Francesca Arfuso, Maria Catena Ferrara, Emanuele Brianti, Gabriella Gaglio

**Affiliations:** 1 Department of Veterinary Sciences, University of Messina, 98168 Messina, Italy; farfuso@unime.it (F.A.); ebrianti@unime.it (E.B.); ggaglio@unime.it (G.G.); 2 Zooprophylactic Institute of Sicily “A. Mirri”, Territorial Area of Barcellona Pozzo di Gotto, 98051 Messina, Italy; maria.ferrara@izssicilia.it

**Keywords:** *Epinephelus marginatus*, nematodes, trematodes, Sicily, Italy, seasonality

## Abstract

**Simple Summary:**

The dusky grouper (*Epinephelus marginatus*) is one of the most expensive species present in the central Mediterranean Sea and the parasite fauna of this species has not been investigated, so far. The aim of the present survey was describing the dusky grouper parasites according to fish size and parasite charge. *E. marginatus* specimens in two groups (cold and warm months) were also divided to establish the relation between parasite fauna and fishing period. According to the results obtained, we can speculate that the infection differences between cold and warm periods could be related to the availability of different prey representing intermediate parasites host. None of the parasites found pose a threat to humans.

**Abstract:**

This study aimed to investigate parasite fauna of *E. marginatus* from the central Mediterranean Sea between Messina and Syracuse. In the present survey; parasite fauna of dusky grouper was investigated for two main reasons: the economic value of this species and the current lack of studies regarding the capture area. Seventy dusky groupers were caught from May 2018 to February 2020. Forty-seven out of the 70 specimens (67.2%) were infected with one or more parasite species. The most abundant species was *Prosorhynchus caudovatus* (42.9%), followed by *Podocotyle temensis* (28.6%), *Didymodiclinus* sp. (18.6%), *Philometra jordanoi* (5.7%), *Anisakis* Type II larvae (5.7%). Higher prevalence of infection of *P. jordanoi* and *Contracaecum* sp. was found in warm months (March to September), while *P. caudovatus* and *P. temensis* were mostly found during cold months. Weight and total length of *E. marginatus* were positively correlated with the parasitic load of *P. jordanoi* and *Didymodiclinus* sp. The different prevalence of parasite infection found between warm and cold months is probably related to the diet of the dusky grouper; which is characterized by mollusks that are intermediate hosts for parasite species found. None of the parasites found in the present survey is responsible for zoonosis

## 1. Introduction

Among marine fish species distributed in warm water, the dusky grouper, *Epinephelus marginatus* (Lowe 1834) is one of the largest top predators in the western Mediterranean littoral ecosystems. Similar to other grouper species, *E. marginatus* shows an ontogenetic change in diet composition and an expansion of the trophic niche, with juveniles feeding primarily on Brachyura crustaceans and adults on cephalopods and fish [1,2]. In addition to its key role in coastal ecosystem food webs, the dusky grouper has a high economic value [3]. *Epinephelus marginatus* shows slow growth, fewer offspring, late maturation, large body size and long lifespan [4]. A decline in Mediterranean dusky grouper stocks has been observed over recent decades [5]. Both behavioral (e.g., site fidelity, inquisitive character) and biological (e.g., hermaphroditism, late sexual maturity, slow growth and longevity) features of this species together with artisanal fisheries and spear-fishing activities are the main factors increasing susceptibility to over-exploitation of *E. marginatus* and are accounted for as the foremost causes of species decline [4].

The occurrence of different parasites has been reported in dusky grouper populations [6,7,8,9,10,11]. In particular, natural outbreaks associated with gnathiid isopod larvae have been observed in wild and captive *E. marginatus*, in some cases associated to significant haematophagia [12,13]. Skin lesions and dermatitis, most likely associated with histozoic parasites, have been described in dusky groupers off the Libyan coast [14]. White capsules, tightly attached to the gills, pseudobranchs and orobranchial cavity with parasites identified as members of the didymozoid family (Trematoda: Didymozoidae) have been reported on wild dusky groupers in the Adriatic Sea [15] and the eastern Atlantic Ocean [16]. More recently, helminthological investigations on marine fish throughout the Mediterranean Sea reported adult philometrid nematodes in gonads of *E. marginatus* [17]. Polinas and co-authors [18] reported white and yellow didymozoid capsules and brown nodules on the gills and pseudobranchs of *E. marginatus* from the western Mediterranean Sea. Widespread occurrence of parasite infections in dusky grouper populations highlights the need for further research into the parasite fauna and infection mechanisms. Despite considerable progress in fish parasitology in recent decades, there are still major gaps in knowledge of parasitic infections affecting wild dusky groupers. Lack of knowledge in this field is related to the complexity of the marine environment. Due to extreme environmental variability, according to scientific literature, it is difficult to improve current knowledge on parasite control in wild fish. Therefore, the aim of the current survey was to characterize the gastrointestinal and gill parasite fauna of *E. marginatus* caught in the central Mediterranean Sea (Southern Italy). Moreover, as the helminth fauna of the dusky grouper is unknown, parasitic infection based on *E. marginatus* development processes was investigated.

## 2. Materials and Methods

### 2.1. Fish Sampling

**Figure 1:**
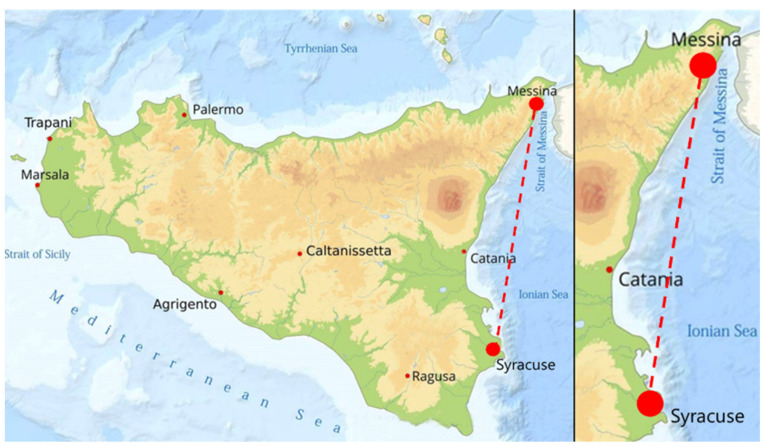
Study area between Messina and Syracuse.

From May 2018 to February 2020, 70 internal organs (stomach, intestine, liver, spleen) and gills of dusky groupers were collected from different fish markets or seized (due to illegal fishing, in particular regarding size) during official checks by veterinarians on the east cost of Sicily (Southern Italy). All the fish were caught in the Central Mediterranean Sea (FAO area 37.2.2) (Figure 1), along the coast between Messina and Syracuse, an area characterized by the presence of cliffs and beaches, with a sandy and rocky seabed, sudden variations of sea depth and a mean water temperature of 20 °C during warm periods and 17 °C during cold periods. Fish were identified with a consecutive number, and the weight (PBA220, Mettler Toledo, accuracy of 1 g) and total length (with an accuracy of 0.1 cm) recorded during sampling. After collection, fish were immediately stored at +4 °C and transferred to the laboratory of Parasitology and Parasitic Diseases, University of Messina and examined within 12 h. The collected specimens were split into two groups according to the capture period, namely warm months (23 specimens), for those collected from March to September, and cold months (47 specimens), from October to April. At the same time, the specimens were divided into size classes according to the criteria defined by Reῆones et al. [2] as follows: Class I (<30 cm TL); Class II (30–45 cm TL); Class III (45–60 cm TL); Class IV (>60 cm TL).

### 2.2. Anatomopathological and Parasitological Examination

A careful macroscopic examination was performed to highlight any lesions present. Examination of gills and internal organ surface was performed for each fish to investigate the presence of parasites with the aid of a stereomicroscope (SteREO Discovery.V12 Zeiss, Jena, Germany). All gastrointestinal organs were inspected for helminths with the total worm count technique (TWC). The parasites collected were stored in 70% ethanol until morphological identification. Parasites were stained, clarified in glycerin for 24 h, mounted and then identified with keys [2,19,20,21,22,23,24]. Trematods were stained with classic Semichon’s carmine red technique [25], modified according to requirements. A biopsy of gills and all organs was performed for microparasite presence. All morphological analyses were performed under an optic microscope (Axioskop 2 plus Zeiss), and all pictures were taken with a digital camera (Axiocam Mrc Zeiss) and a digital system (Axiovision Zeiss).

### 2.3. Statistical Analysis

For each parasite species, the epidemiological indices of infection as prevalence (P, %), mean abundance (MA) and mean intensity (MI) were calculated according to Bush et al. [26], and Pearson’s chi-square analysis applied to evaluate differences of most frequent parasite species in *E. marginatus* specimens between warm and cold months. Pearson’s correlation coefficients were computed to evaluate the relationship between the biometric data (weight and total length) of *E. marginatus* and the prevalence of infection of each parasite species. A linear regression model (y = a + bx) was applied to determine the degree of correlation between these parameters during the study period. Level of significance was set at *p* values < 0.05. Statistical analyses were performed using the software GraphPad Prism version 5.1 (GraphPad Software, San Diego, CA, USA).

## 3. Results

Examined *E. marginatus* specimens (3 males and 67 females) weighed from 280 g to 8 kg and had a total length (TL) ranging from 18 to 80 cm, divided as following: Class I (N = 21 fish); Class II (N = 26 fish); Class III (N = 11 fish); Class IV (N = 12 fish,). Parasites were isolated from several organs including the intestine, stomach, gills and gonads. Specifically, 47 out of the 70 *E. marginatus* specimens (67.2%) were positive for one or more parasite species or taxa (Table 1).

**Figure 2:**
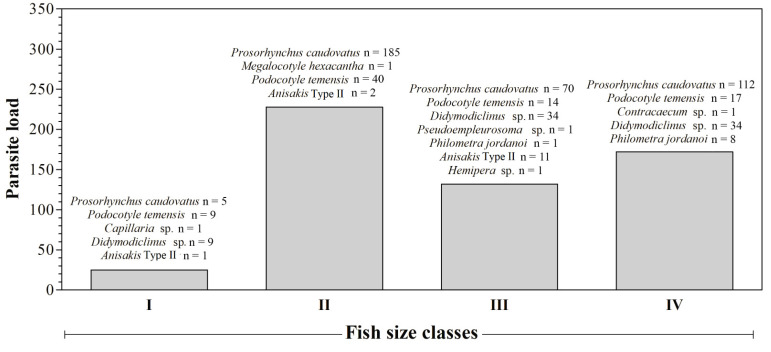
Number of parasites per species according to different size classes.

**Figure 3:**
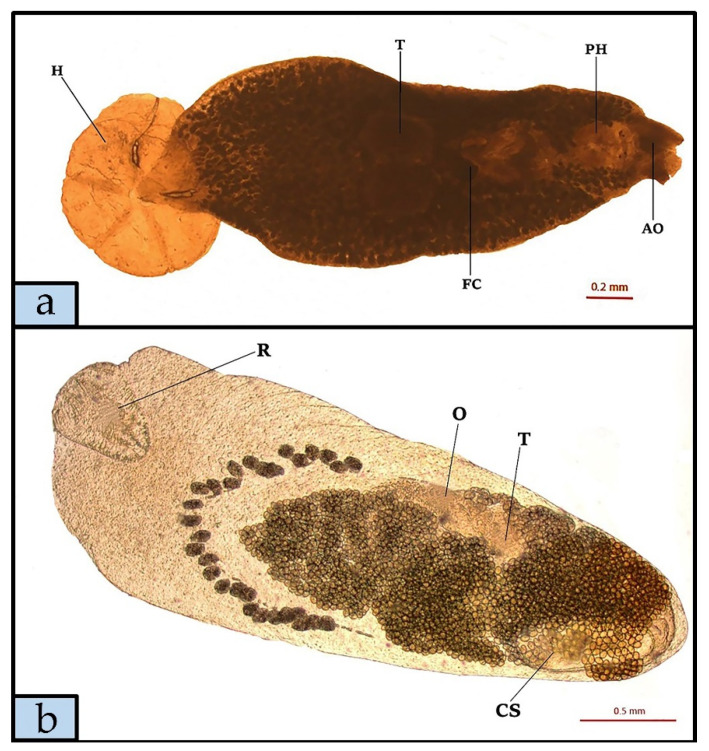
*Megalocotyle hexacantha* (**a**) specimen isolated from *Epinephelus marginatus* gills, glycerine diaphanization (H, Haptor; T, Testis; FC, Internal fertilization chamber; PH, Pharynx; AO, Anterior fixation organ) and *Prosorhynchus caudovatus* specimen (**b**) isolated from the *E. marginatus* stomach, glycerine diaphanization (R, Rhynchus; O, Ovary; T, Testis; CS, Cirrus sack).

**Figure 4:**
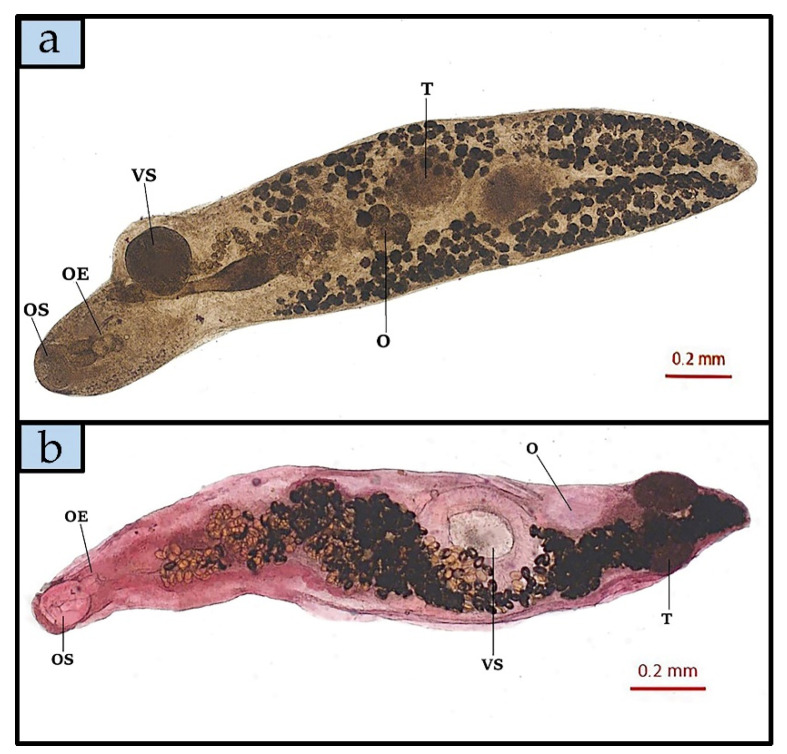
*Podocotyle temensis* specimen (**a**), glycerine diaphanization (O, Trilobal ovarian tissue; T, Testis; OE, Oesophagus; OS, Oral sucker; VS, Ventral Sucker) and *Hemipera* sp. specimen (**b**), after Semichon’s carmine red technique staining (O, Ovary; T, Testis; OE, Oesophagus; OS, Oral sucker; VS, Ventral Sucker), both isolated from *Epinephelus marginatus* intestine.

No microparasites were found by biopsy. *Prosorhynchus caudovatus* and *P. temensis* showed the highest rate of infection compared to the other parasite species found, also according to analysis of the size classes (Figure 2). According to species descriptions by Yamaguti [19] and Polinas et al. (2018) [18], three flukes identified as *Didymodiclinus* sp., *Pseudoempleurosoma* sp. and *Megalocotyle hexacantha* were found in gills (Figure 3a). Morphologic features of other parasites, observed after diaphanization, allowed the identification of the gastrointestinal flukes *Prosorhynchus caudovatus* (Figure 3b), *Podoctyle temensis*, *Hemipera* sp. (Figure 4a,b), and the nematode *Philometra jordanoi*, *Anisakis* sp. type II larvae, *Capillaria* sp. and *Contracaecum* sp.

Gross lesions in the coelomatic organs of *E. marginatus* specimens attributable to parasite infection were not observed, and macroscopic gonadal alterations resulting from the presence of *P. jordanoi* (one to five specimens per fish) or gill damage caused by *Didymodiclinus* sp. were not found.

As reported in Table 1, statistical evaluation of the data showed a dynamic infection level in *E. marginatus* specimens caught throughout the study period. In particular, the most prevalent parasite species *P. caudovatus* (χ^2^ = 7.55, *p* = 0.006) and *P. temensis* (χ^2^ = 7.61, *p* = 0.005) were mostly found in cold months (October to March). The other parasite species showed no statistically significant difference in the prevalence of infection between warm and cold months. Biometric data, including weight and TL of *E. marginatus* investigated in the current survey, were positively correlated with the parasitic load of *P. jordanoi* and *Didymodiclinus* sp. (Table 2).

**Figure 5:**
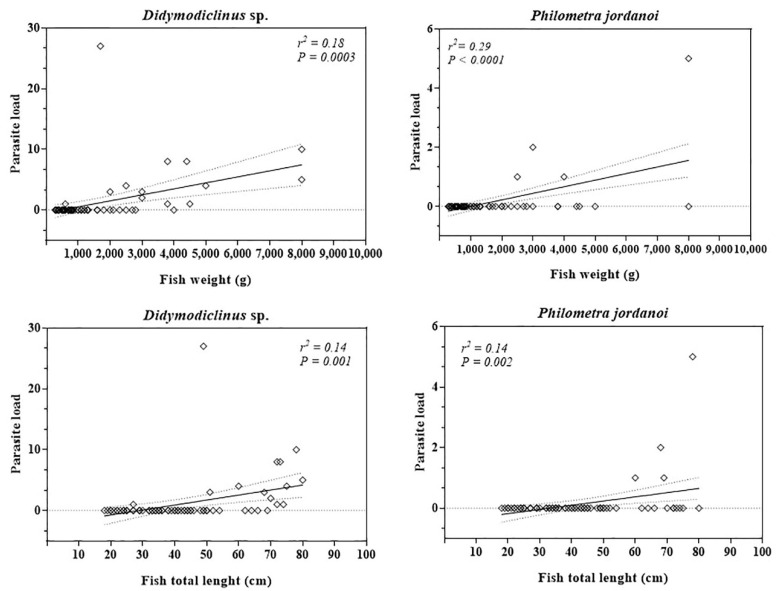
Linear regression values obtained between biometric data (weight and length) of *Epinephelus marginatus* and prevalence of infection of *Didymodiclinus* sp. and *Philometra jordanoi*.

The results of Pearson correlation were confirmed by the linear regression model (Figure 5).

## 4. Discussion

The present survey provides an overview of the parasite fauna of *E. marginatus* from a central Mediterranean Sea population that had not been investigated previously. *Prosorhynchus caudovatus* was the most prevalent species found in the current survey. Interestingly, this species has not been reported in *E. marginatus* of the Mediterranean area, so far. Only a morphological description of *P. caudovatus* isolated from one specimen of *E. marginatus* caught along the South African coast [27], and in two *Epinephelus goreensis* and in four *Lutjanus maltzani* caught along the Ghanaian coast, have been reported [28].

The species *P. temensis* found here with a prevalence of 28.6% represents the first report of this parasite in *E. marginatus* from the investigated area. Previously, this species was reported in a dusky grouper along Corsican coasts, where 22 specimens of *P. temensis* were found in the pyloric caeca of one *E. marginatus* [20]. The gonads of 4 out of the 70 studied fish were infected with one to five specimens of *P. jordanoi*, but no gross organ alterations were observed. This finding is different to that reported by Marino et al. (2016) [29] in *Pagellus erythrinus* massively infected by gravid *Philometra filiformis*; probably, the smaller size of subgravid females of *P. jordanoi* found in dusky grouper could account for this difference. Philometrid nematodes have been reported with a prevalence of 22% in *E. marginatus* gonads and, despite the difficulty of extraction and identification, according to Lòpez-Neyra [30] a male specimen was identified as *P. jordanoi*.

Didymozoidae species found in the current survey was identified as *Didymodiclinus* sp. Although this is the first report of *Didymodiclinus* sp. from the central Mediterranean Sea, the presence of this species had previously been reported in an *E. marginatus* specimen caught off Majorca Island (Western Mediterranean Sea) [18]. 

The *Pseudempleurosoma* sp., *Contracaecum* sp., *M. hexacantha*, *Capillaria* sp., *Hemipera* sp. and larvae of *Anisakis* type II found in specimens, even though in low numbers, represent the first report in *E. marginatus* from the central Mediterranean Sea so far.

Analysis of the data highlighted a very high prevalence for infections with *P. caudovatus* and *P. temensis*, whose life cycle depends on an intermediate host presence. Although current knowledge on the life cycle of these flukes is limited, the high prevalence of infection of *P. caudovatus* and *P. temensis* found here suggests an abundance and/or preference of intermediate hosts of these parasite species in the diet of the dusky grouper.

The most frequently found parasitic species mostly found during warm months were *Philometra jordanoi* and *Contracaecum* sp., whereas *P. caudovatus* and *P. temensis* showed a higher prevalence of infection during cold months. Differences found in parasite infections between warm and cold months are probably related to the diet and availability of food source. According to Reῆones et al. [2], juvenile *E. marginatus* specimens (<30 cm TL) caught in the Mediterranean Sea feed mainly on Brachyura (46% of stomach contents), in medium size specimens, between 30 and 45cm, and from 45 to 60 cm (sub-adult stage) the diet is mainly represented by cephalopods (40% of stomach contents), in adult specimens, >60 cm, the diet is mainly represented by teleost. Some of these varied organisms (e.g., Crustaceans, small fish, cuttlefish and octopus) comprising the diet of *E. marginatus* could potentially act as intermediate hosts for identified endo- and/or ecto-parasite species in this study [1,2]. Due to the size of the most parasitized fish, it could be speculated that mollusks represented the main source of nourishment of the *E. marginatus* analyzed [2], making them possible intermediate hosts of the flukes and nematodes found. Moreover, the data collected in the present study showed that weight and total length of *E. marginatus* are strongly correlated with *Didymodiclinus* sp. and *P. jordanoi* infection suggesting that fish size and, therefore, age, are variables worthy of investigation for the parasitic fauna of the dusky grouper as well as of other fish species of commercial interest. A possible explanation for the positive correlation between fish size and presence of *Didymodiclinus* sp. and *P. jordanoi* once again falls on the diet of *E. marginatus* as, being young fish, they are unable to prey on intermediate hosts of these parasites, e.g., mollusks and other fish; this strong positive relationship between parasite abundance and fish size has previously been reported by Polinas et al. [18]. 

There are still many gaps in the life cycle of the parasite species infesting dusky grouper and, thus, justifications for the results of the present study are in part speculative. According to the findings obtained, the dusky grouper of the investigated sea area may be regarded as a fish species that is “poor” in parasites regarding both abundance and variety of parasite species. According to Adroher-Auroux and Benítez-Rodríguez [31], there are only two reports of *Anisakis* type II in mammals, both in laboratory rats during an experimental infection. For these reasons, it is impossible to suspect a possible zoonotic risk due to dusky grouper consumption. Nevertheless, the present survey allowed us to describe the presence of 10 parasite species in *E. marginatus* specimens caught in Sicilian seawaters which helps to better understand the parasite fauna of this fish species and the potential hazards posed, both to the host and to human consumers.

## 5. Conclusions

This investigation improves current knowledge on the parasitic fauna of *E. marginatus*, the most valued serranid of the fish population inhabiting Mediterranean coasts. Moreover, the current survey showed that the parasite species found and identified do not pose any public health concern.

## Figures and Tables

**Table 1:** Prevalence (P, %), Mean abundance (MA) and mean intensity (MI) and infection site (Stomach = S, Intestine = I, Gills = G, Gonads = GO) of infection of parasite species retrieved in dusky grouper specimens.

	P (%)			MA			MI		
Parasites with Indirect Life Cycle	Total	Warm Months	Cold Months	Total	Warm Months	Cold Months	Total	Warm Months	Cold
Infection Site									Months
*Prosorhynchus caudovatus* (S, I)	42.9	30.4 ^A^	48.9	5.32	4.39	5.77	7.90	7.77	8.47
*Podocotyle temensis* (S, I)	28.6	17.4 ^A^	34.0	1.14	0.43	1.49	1.70	0.77	2.19
*Hemipera* sp. (I)	1.4	0	2.1	0.01	0	0.02	0.02	0	0.03
*Didymodiclinus* sp. (G)	18.6	7.4	19.1	1.10	0.95	1.17	1.63	1.69	1.72
*Philometra jordanoi* (GO)	5.7	13.0	2.1	0.40	0.34	0.02	0.19	0.61	0.03
*Anisakis* sp. type II (S, I)	5.7	8.7	4.3	0.21	0.48	0.06	0.31	0.84	0.09
*Capillaria* sp. (S)	1.4	0	2.1	0.01	0	0.02	0.02	0	0.03
*Contracaecum* sp. (S)	1.4	4.3	0	0.10	0.04	0	0.02	0.08	0
	P (%)			MA			MI		
**Parasites with Direct Life Cycle**	**Total**	**Warm Months**	**Cold Months**	**Total**	**Warm Months**	**Cold Months**	**Total**	**Warm Months**	**Cold Months**
**Infection Site**									
*Pseudempleurosoma* sp. (G)	1.4	4.3	0	0.01	0.04	0	0.02	0.08	0
*Megalocotyle hexacantha* (G)	1.4	4.3	0	0.01	0.04	0	0.02	0.07	0

^A^ Significant difference found (*p* < 0.05).

**Table 2:** Correlation between biometric data of *Epinephelus marginatus* and parasites’ prevalence.

	**Biometric Data of *Epinephelus marginatus***	
**Parasite Species**	***Weight (*g*)***	***Total Length (*cm*)***
*Pseudempleurosoma* sp.	r = 0.07, *p* = 0.56	r = 0.13, *p* = 0.27
*Megalocotyde hexacantha*	r = −0.02, *p* = 0.86	r = 0.03, *p* = 0.82
*Prosorhynchus caudovatus*	r = 0.08, *p* = 0.52	r = 0.20, *p* = 0.10
*Didymodiclinus* sp.	**r = 0.42, *p* = 0.0003**	**r = 0.37, *p* = 0.001**
*Philometra jordanoi*	**r = 0.53, *p* < 0.0001**	**r = 0.14, *p* = 0.001**
*Anisakis* sp. type II	r = 0.06, *p* = 0.65	r = 0.12, *p* = 0.32
*Podocotyle* sp.	r = 0.02, *p* = 0.88	r = 0.12, *p* = 0.32
*Hemipera* sp.	r = 0.01, *p* = 0.93	r = 0.06, *p* = 0.65
*Capillaria* sp.	r = −0.07, *p* = 0.54	r = −0.10, *p* = 0.40
*Contracaecum* sp.	r = 0.11, *p* = 0.37	r = 0.19, *p* = 0.12

Significant correlations (*p* < 0.05) are indicated in bold letters.

## Data Availability

The data presented in this study are available on request from the corresponding author.
